# Case-control accuracy study for TOY8 digital developmental screening tool for detecting delays among children aged 3–5 years

**DOI:** 10.3389/fped.2025.1706162

**Published:** 2025-12-02

**Authors:** Teck-Hock Toh, Yvonne Mei-Fong Lim, Jeffrey Soon-Yit Lee, Wai-Nam Chan, Zhen-Ying Low, Kamilah Dahian, Siok-Cheng Chew, Sheamini Sivasampu, William Kian-Boon Law, Amar-Singh HSS

**Affiliations:** 1 Clinical Research Centre, Sibu Hospital, Ministry of Health Malaysia, Sibu, Malaysia; 2 Department of Paediatrics, Sibu Hospital, Ministry of Health Malaysia, Sibu, Malaysia; 3 National Early Childhood Intervention Council, Kuala Lumpur, Malaysia; 4 Faculty of Medicine, Nursing & Health Sciences, SEGi University, Kota Damansara, Malaysia; 5 Institute for Clinical Research, National Institutes of Health, Ministry of Health Malaysia, Setia Alam, Malaysia; 6 Department of Paediatrics, Kapit Hospital, Ministry of Health Malaysia, Kapit, Malaysia; 7 Department of Early Childhood Education, Methodist Pilley Institute, Sibu, Malaysia

**Keywords:** screening, digital tool, developmental delay, preschool children, TOY8, Malaysia

## Abstract

**Background:**

Developmental delays affect up to 18% of children worldwide, particularly in disadvantaged populations. Early identification is critical; however, existing tools are often resource-intensive, language-dependent, and unsuitable for large-scale use in low- and middle-income countries. TOY8 is a smartphone-based, play-oriented developmental screening tool developed in Malaysia for children aged 3–5 years, available in Malay and English.

**Purpose:**

To validate TOY8 against the Griffiths Scales of Child Development, 3rd Edition (Griffiths III), determine optimal cut-offs, and assess parental perceptions of feasibility and acceptability.

**Methods:**

We conducted a case-control study in Sarawak, Malaysia. Participants underwent TOY8 screening followed by Griffiths III assessment. Screening performance was evaluated using sensitivity, specificity, likelihood ratios, and receiver operating characteristic (ROC) analyses. Optimal cut-offs were derived by maximizing sensitivity while maintaining specificity at ≥0.6. Parental perceptions were measured using questionnaires.

**Results:**

We recruited 127 children (64 with developmental delay, 63 without). TOY8 demonstrated good sensitivity (0.77) for detecting any developmental delay and higher sensitivity for severe delay (0.84). Cognitive, speech-language, and fine motor domains demonstrated excellent discrimination (AUC 0.82–0.84), but lower sensitivity for gross motor (0.41–0.54) and personal-adaptive domains (0.59–0.64). Refined domain-specific cut-offs (ROC: 44–50) improve screening accuracy. Parents rated TOY8 highly: 98.4% found it easy/very easy to use, 99.2% useful, and 96.9% acceptable.

**Conclusion:**

TOY8, the first digital developmental screening tool validated in Malaysia, demonstrated good accuracy, particularly in domains predictive of school readiness. Its brevity, ease of use, and strong parental acceptability support its feasibility for community and preschool settings. TOY8 offers a scalable solution for early detection in resource-limited contexts, directly advancing United Nations Sustainable Development Goal (SDG) 3 on health and well-being, and SDG 10 on reducing inequalities by improving access to developmental screening in underserved populations.

## Introduction

Early childhood is a period of rapid physical, cognitive, motor, and socio-emotional development, typically monitored through developmental milestones that provide benchmarks for expected achievements at different ages (1). Inability to achieve age-based developmental milestones can signal underlying developmental disorders, or global developmental delay, defined as a significant lag in two or more domains (2, 3). Reported prevalence is as high as 16% to 18% and appears to have increased, largely reflecting improved awareness and detection rather than a true rise in incidence (4). Rates are higher in disadvantaged populations, underscoring the importance of timely detection and intervention (4, 5).

In Malaysia, developmental surveillance is routinely provided only until 18 months of age, integrated with the Ministry of Health's immunization schedule. Between the ages of two and six, children generally do not receive structured surveillance, while private sector services are limited and costly (6). On the other hand, some disorders often become more evident in the preschool years, including developmental language disorder, autism spectrum disorder, intellectual disability, attention deficit hyperactivity disorder, and specific learning disorders (e.g., dyslexia) (3, 6). This gap mirrors international concerns, where structured developmental surveillance after age three is inconsistently practiced (3).

Various instruments are available for developmental screening and assessment. Common screening tools, such as the Brigance Early Childhood Screen, Schedule of Growing Skills, and Denver Developmental Screening Test, rely on parental or teacher reports, direct observation, or both. More comprehensive assessments, including the Griffiths Scales of Child Development, 3rd Edition (Griffiths III), and the Bayley Scales of Infant and Toddler Development, provide in-depth evaluations across multiple domains. However, these assessments are resource-intensive, requiring trained healthcare professionals and lengthy administrative time, which limits their feasibility for community screening, especially in low- and middle-income countries (LMIC) with limited specialist services. Furthermore, few culturally adapted tools have been validated for children under five years in such contexts (7). Parent-report instruments, such as the Child Behavior Checklist and the Gilliam Autism Rating Scale, are easier to use but focus largely on behavioral domains and also require professional interpretation. Although effective in high-risk groups, they are not practical for universal screening. Accordingly, a pressing need remains for an accessible and scalable tool designed to be used with minimal specialized expertise. Given recommendations that developmental surveillance continue through preschool years, there is a clear need for practical tools applicable to 3- to 5-year-olds in community and preschool settings (3).

TOY8 is a digital, play-based developmental screening tool developed in Malaysia for children aged 3–5 years, available in both Malay and English. Created by an ex-Nintendo game developer, it uses artificial intelligence (AI) to analyze children's interactions with engaging, game-like tasks that assess five developmental domains: gross motor, fine motor, speech-language, cognitive, and personal-adaptive skills. Designed for use by trained non-healthcare professionals on a smartphone, TOY8 provides an immediate, domain-specific developmental profile to support early identification and communications with parents (8). This approach bridges technology, play, and accessibility, enabling culturally appropriate developmental screening in community and preschool settings.

Early internal validation of TOY8 involving 9,000 Malaysian children demonstrated good psychometric reliability and cross-language consistency, reflecting potential as a scalable community tool (9). However, formal validation against a gold-standard developmental assessment is necessary before widespread adoption. This study, therefore, aimed to evaluate the sensitivity, specificity, and discrimination of TOY8 compared with Griffiths III for screening developmental delays in children. In addition, we also aimed to establish optimal cut-off scores for detecting developmental delays and to evaluate their feasibility, acceptability, and usability in community settings.

By addressing a critical gap in preschool developmental surveillance, this work aligns with the United Nations Sustainable Development Goals (SDGs) (10). It supports SDG 3, ensuring healthy lives and promoting well-being for all at all ages, and SDG 10, reducing inequalities by expanding access to early identification and intervention in underserved populations (11). Findings from this study will inform the role of TOY8 as a culturally adapted, practical, and scalable tool for early detection of developmental delays in Malaysia and other LMICs.

## Materials and methods

### Study design and reporting

This prospective, case-control validation study employed stratified random sampling of children, enabling evaluation of TOY8's accuracy across a broad spectrum of developmental delays (12). Recruitment was conducted between April and August 2024. Parents provided written informed consent. All children first completed TOY8, followed by the Griffiths III as the reference standard. The study was approved by the Medical Research and Ethics Committee, Ministry of Health Malaysia [NMRR ID-24-00262-TV1 (IIR)]. It was conducted in accordance with the principles outlined in the Declaration of Helsinki. The manuscript is reported in accordance with the Standards for the Reporting of Diagnostic Accuracy (STARD) (13).

### Participants and sites

Children aged 3 years 0 months to 5 years 11 months (calculated from date of birth to the date of Griffiths III assessment) were eligible. A case was defined as a child with a known developmental delay identified by site investigators at the Lau King Howe Memorial Children's Clinic @ Agape Centre, a community-based child development clinic. A control was a child without developmental, behavioral, or learning concerns, who might be siblings to a child attending the clinic, visited the Mother-and-Child Health Clinic (MCH), general pediatric clinics at Sibu Hospital, or attended local preschools.

### Eligibility criteria

All children could understand and communicate in simple Malay or English. Cases are children with developmental disabilities confirmed by a pediatrician, including autism spectrum disorder (DSM-5 Level I or II), or global developmental delay (significant delay in ≥2 developmental domains). Children unable to ambulate unassisted (either because of cerebral palsy, spina bifida, limbs abnormalities, muscular dystrophy, or any other developmental disabilities), has confirmed hearing impairment (except for those not requiring hearing aids), and/or visual impairment affecting abilities to read and not corrected by spectacles, and those too hyperactive or non-cooperative in completing the tests, were excluded. Children with obvious genetic conditions (e.g., Down syndrome or other genetic disorders) were also excluded.

### Developmental screening tool

TOY8 was administered on a smartphone, it engages children with a cartoon character through interactive tasks such as answering questions, drawing, or stacking. Screenings were supervised by trained non-healthcare personnel, such as early childhood education teachers with knowledge and experience in child development. Minimal training is required to use the tool effectively. TOY8 evaluates five domains: cognitive, speech-language, fine motor, gross motor, and personal-adaptive behavior. A domain score <40 indicated developmental delay. Further details on the TOY8 tool are described in the article by Wo et al (9). Parents also completed a questionnaire component of TOY8 on gross motor and social-emotional skills.

### Developmental reference assessment test

Griffiths III measures child development from birth to six years across five subscales: Foundations of Learning, Language and Communication, Eye-Hand Coordination, Personal–Social–Emotional, and Gross Motor (14). Standardization of the Griffiths Mental Development Scales—Extended Revised was conducted by the Association for Research in Infant and Child Development in 2015 on samples from the United Kingdom and Ireland (15). In this study, Griffiths III was administered by trained research nurses or a pediatrician under the supervision of the first author (16). Children scoring below the 10th centile in any subscale were categorized as having mild-moderate delay; those below the 3rd centile were categorized as severe delay.

For validation, TOY8 domains were mapped against Griffiths III subscales:

- Cognitive → Foundations of Learning
- Speech-Language → Language and Communication
- Fine Motor → Eye and Hand Coordination
- Gross Motor → Gross Motor
- Personal-Adaptive Behavior → Personal-Social-Emotional

### Reliability testing

To assess inter-rater reliability, a subset of 10 children (from those with and without a history of developmental delay) underwent Griffiths III with simultaneous scoring by two assessors. While a child was undergoing the Griffiths III test, a second rater sat and observed the assessment without interruption and scored the assessment. The intraclass correlation coefficient was 0.99 (95% CI: 0.998, 0.999), indicating excellent agreement. Bland-Altman analysis showed a mean difference of 0.01 (95% CI: −0.884, 0.894), with a maximum raw score difference of two points, suggesting no systematic bias (17).

Children in the control group identified with developmental delays on Griffiths III were offered a free follow-up assessment by a consultant pediatrician at the same clinic. Those without concern or delay continued routine developmental surveillance at their MCH clinics.

### Sample size calculation, sampling and study procedure

In this study, the sample size was estimated to be at least 124 subjects, in a 1:1 ratio for children with and without a known history of developmental delay. The calculation was based on the method by Burderer et al. (18). The total sample size required was 124 children, with 62 each for those with and without history of delay. Details of the sample size calculation can be found in the Supplementary File S1.

To ensure demographic representation, sampling was stratified by age and sex. Details of the approximate distributions are summarized and available in the Supplementary File S1. All eligible records were identified from patient registration books, categorized by age and gender strata, and randomly sampled.

Each child attended at least two visits. At enrollment (days −14 to day 0), procedures included obtaining informed consent, screening for eligibility criteria, and collecting demographics and medical history. TOY8 screening was generally conducted on the same day as enrolment, though in some instances it occurred within one week [*n* = 15 (21.1%) for those with a history of delay; *n* = 8 (12.5%) for those without]. Because both TOY8 and Griffiths III measure developmental status at a specific age, the interval between the two tests was kept short. Griffiths III testing was performed at least seven days (and up to 14 days) after TOY8 to minimize potential carry-over effects, such as improved task familiarity, motor coordination, or memory recall from the first test. On the TOY8 screening day, parents were also surveyed on their perceptions of the tool's ease of use, usefulness, and acceptability.

If a child was unwell on the test day (e.g., fever, acute respiratory illness, recent seizure, or medication use within 24 h), testing was postponed. Parents were allowed to be present during both TOY8 and Griffiths III sessions, but were not permitted to intervene, prompt, or correct the child. Those who intervened were cautioned or asked to leave the room. Families received a one-off reimbursement of MYR30 (about USD7) for transport per visit involving TOY8 and Griffiths III. Assessment reports were provided free of charge. Data from participants who withdrew were not included in the analysis.

### Statistical analysis

Analyses were conducted using R version 4.4.1 (19), with statistical significance set at *p* < 0.05. Missing data were minimal; only ethnicity data were incomplete (2%). For parental education, the highest level attained by either parent was recorded. Sensitivity and specificity of the TOY8 were reported, and 0.7 was the accepted minimum sensitivity (20). To facilitate interpretation of screening results at an individual child level, likelihood ratios were computed in addition to sensitivity and specificity. The positive likelihood ratio (LR+) gave the probability of the outcome given a positive score on the screening tool. The absolute value of the LR+ from 1 to 2 indicated a minimal probability of the outcome, 2–5 a small probability, 5–10 a moderate probability, and ≥10 indicated a large, conclusive probability (21).

Receiver operating characteristic (ROC) curves illustrated the trade-off between sensitivity and specificity. The area under the ROC curve (AUC) was used to quantify discriminative performance: 0.7–0.8 acceptable, 0.8–0.9 excellent, and >0.9 outstanding (22, 23). Finally, optimal cut-off scores for the TOY8 subscales were determined by maximizing sensitivity while maintaining a specificity of ≥0.6. This criterion was chosen to reflect the screening purpose of TOY8, the high expected prevalence of developmental delay in the community, and the associated costs of missed cases (24, 25).

## Results

### Participants

Of 135 eligible children, 127 (94.1%) completed the TOY8 and Griffiths III assessments. Eight participants were excluded due to incomplete study procedures (seven with history of developmental delays, one without): unable to complete the Griffiths III because of hyperactivity and/or lack of cooperation (*n* = 5), violation of eligibility criteria (*n* = 1), and withdrawal due to transport issues (*n* = 2). The final cohort had a median age of 4.5 years (range 3.1–5.96 years); 51.2% (*n* = 65) were boys. The case-control design yielded equal representation by age strata: 3 years (*n* = 42, 33.1%), 4 years (*n* = 43, 33.9%), and 5 years (*n* = 42, 33.1%). In terms of ethnic distribution, 42.5% (*n* = 54) were Chinese, 26.8% (*n* = 34) were Malays, 21.3% (*n* = 27) were Iban, and the remaining 7.9% (*n* = 10) belonged to other ethnicities. Socio-economic status was balanced between groups, as proxied by parents’ educational level, where 32.3% (*n* = 41) had secondary education or below, 24.4% (*n* = 31) had a Certificate/Diploma, and the remaining 43.3% (*n* = 55) had a Bachelor's degree or higher. TOY8 was substantially quicker to administer than Griffiths III [median 21 min (IQR 17–26) vs. 90 min (IQR 75–105)].

Table 1 presents the socio-demographic characteristics of the children with developmental delay (i.e., cases) and those without delay. All socio-demographic characteristics were similar between the two groups (*p* > 0.05) except for ethnicity and parental education. Notably, 10 children initially recruited as controls were later found to have developmental disorders on Griffiths III**,** highlighting undetected problems within the community sample.

**Table 1:** Baseline characteristics of children with and without a known history of developmental delay.

Characteristic	With history of delay *N* = 64^a^	Without history of delay *N* = 63^a^	*p*-value^b^
Age (year)			0.9
Median (Q1, Q3)	4.49 (3.74, 5.18)	4.47 (3.75, 5.32)	
Min, Max	3.14, 5.95	3.05, 5.96	
Age category			0.9
3–<4	21 (32.8%)	21 (33.3%)	
4–<5	23 (35.9%)	20 (31.7%)	
5	20 (31.3%)	22 (34.9%)	
Sex			>0.9
Female	31 (48.4%)	31 (49.2%)	
Male	33 (51.6%)	32 (50.8%)	
Ethnicity			0.030
Malay	21 (32.8%)	13 (20.6%)	
Chinese	19 (29.7%)	35 (55.6%)	
Iban	17 (26.5%)	10 (15.9%)	
Others	6 (9.4%)	4 (6.4%)	
Unknown	1 (1.6%)	1 (1.6%)	
Birth order			0.10
First	29 (45.3%)	28 (44.4%)	
Second	13 (20.3%)	22 (34.9%)	
Third and above	22 (34.4%)	13 (20.6%)	
Number of siblings			0.7
0	12 (18.8%)	11 (17.5%)	
1	27 (42.2%)	31 (49.2%)	
2 or more	25 (39.1%)	21 (33.3%)	
Parent's educational level^c^			<0.001
None/Primary	0 (0%)	1 (1.6%)	
Secondary	26 (40.6%)	14 (22.2%)	
Certificate/Diploma	21 (32.8%)	10 (15.9%)	
Bachelor's degree or higher	17 (26.6%)	38 (60.3%)	
Parents’ marital status			0.2
Married	63 (98.4%)	59 (93.7%)	
Single/Separated/Widowed	1 (1.6%)	4 (6.3%)	
Medical history^d^			0.2
Language disorder	18 (28.1%)	8 (12.7%)	
Autism spectrum disorder	17 (26.6%)	0 (0%)	
Intellectual disability	10 (15.6%)	2 (3.2%)	
Severe language disorder with borderline intellectual disability	7 (10.9%)	0 (0%)	
Global developmental delay	9 (14.1%)	0 (0%)	
Speech/language delay	3 (4.7%)	0 (0%)	
Not present	0 (0%)	53 (84.1%)	
Duration of Toy8 assessment (min)			0.001
Median (Q1, Q3)	23 (18, 31)	20 (16, 23)	
Min, Max	13, 74	12, 45	
Duration of Griffiths III assessment (min)			0.6
Median (Q1, Q3)	90 (75, 105)	90 (75, 105)	
Min, Max	45, 135	60, 130	

a *n* (%).

b Wilcoxon rank sum test; Pearson's Chi-squared test; Fisher's exact test.

c Highest educational level attained between parents.

d Medical history related to delay for control group was determined from Griffiths II assessment in this study.

### TOY8 scores in children with and without developmental delays

The study population was diverse with respect to the number of subscales affected by delay on the Griffiths III assessments. Thirty-one percent (*n* = 39) of the children had no delay, 21.3% had a developmental delay in only one subscale, 12.6%, 8.7%, 11.0% and 15.7% had a delay in two, three, four, and five subscales, respectively. Table 2 shows the percentage of children with positive TOY8 scores for any developmental delay. Delays were most common in Speech-Language (66.9%, *n* = 85), followed by Personal-Adaptive Behavior, and least in Gross Motor (29.1%, *n* = 37). TOY8 scores were consistently lower among children with delays compared to those without (*p* < 0.001). The largest score gaps were observed in Cognitive (median difference = 23.02), Personal-Adaptive Behavior (21.92), and Speech-Language (18.80).

**Table 2:** TOY8 *T* scores between any developmental delays and no delay groups by subscales.

Subscale	*N* (%) with delays	TOY8 *T* scores^a^		
		Any developmental delays^b^	No delay	*p*-value^c^
Cognitive	45 (35.4%)	28.83 (22.64, 41.77)	51.85 (44.30, 69.99)	<0.001
Speech-Language	85 (66.9%)	34.98 (24.94, 46.33)	53.78 (48.69, 57.89)	<0.001
Fine Motor	30 (23.6%)	30.73 (16.80, 40.45)	47.43 (40.71, 56.95)	<0.001
Personal-Adaptive Behavior	51 (40.2%)	30.94 (18.94, 53.53)	52.86 (41.30, 56.76)	<0.001
Gross Motor	37 (29.1%)	48.05 (44.22, 59.61)	57.59 (52.79, 63.05)	<0.001

a Data presented as median and interquartile range.

b Mann–Whitney U test.

c Assessment based on Griffiths III.

### Screening performance of TOY8

With a cut-off score of 40, TOY8 demonstrated adequate sensitivity (0.77) for detecting mild-to-moderate developmental delay and good sensitivity (0.84) for severe delays (Table 3). Cognitive and Fine Motor domains demonstrated acceptable to good sensitivity for mild-to-moderate delays (0.71 and 0.73) and severe delays (0.80 and 0.79).

**Table 3:** Screening performance of TOY8 for mild-to-moderate delays and severe delays.

Severity of delays and subscales	Sensitivity	Specificity	LR+	LR-
Mild-to-moderate delays				
Any subscale	0.77	0.67	2.32	0.34
Cognitive	0.71	0.82	3.89	0.35
Speech language	0.64	0.88	5.34	0.41
Fine motor	0.73	0.76	3.09	0.35
Personal-adaptive behavior	0.59	0.84	3.73	0.49
Gross motor	0.41	0.82	2.28	0.72
Severe delays				
Any subscale	0.84	0.65	2.43	0.24
Cognitive	0.80	0.76	3.37	0.26
Speech-language	0.76	0.89	7.19	0.27
Fine motor	0.79	0.72	2.84	0.29
Personal-adaptive behavior	0.64	0.82	3.64	0.43
Gross motor	0.54	0.83	3.10	0.56

LR+, positive likelihood ratio; LR−, negative likelihood ratio.

**Figure 1:**
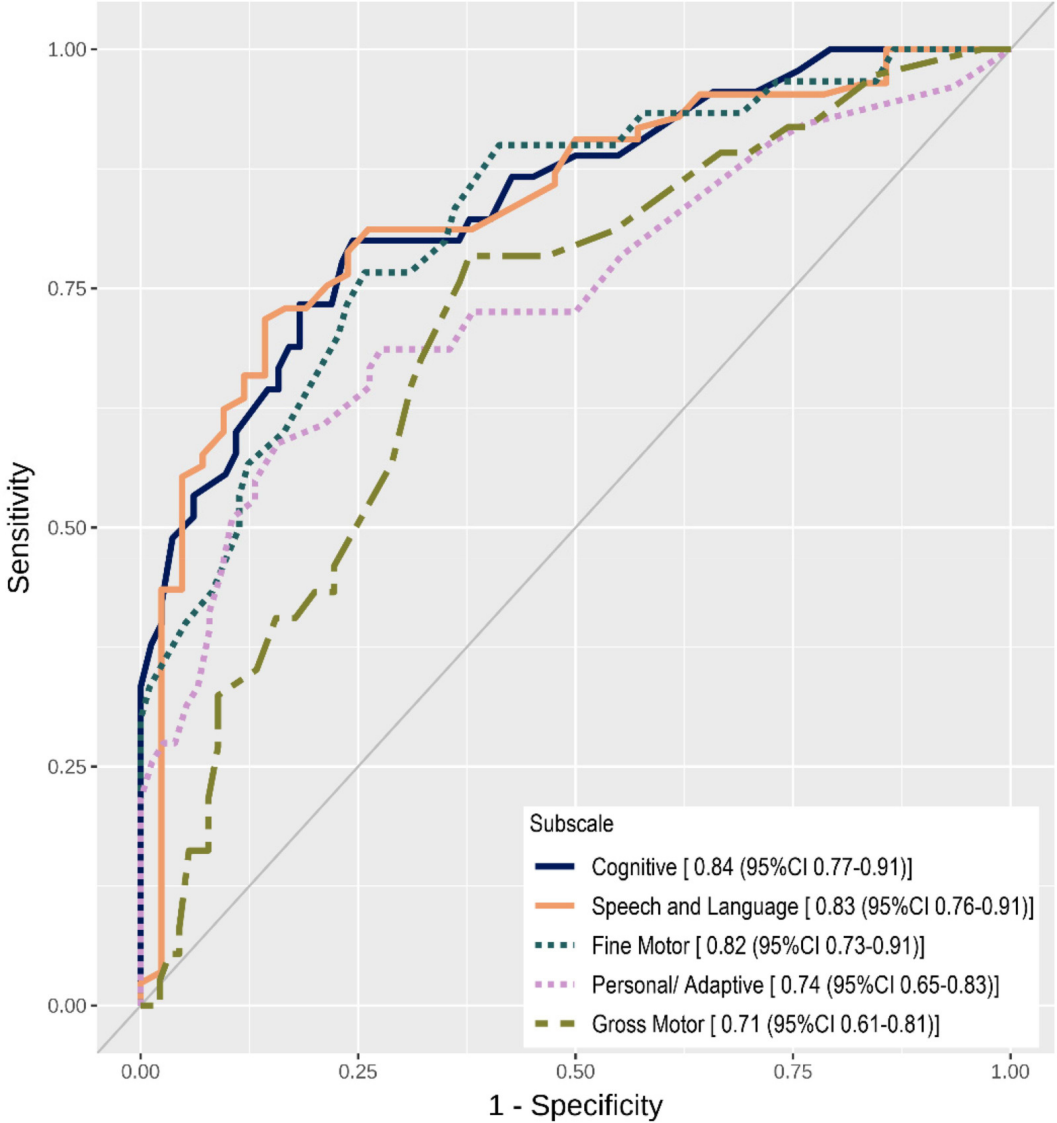
Receiver operating characteristics curve by subscale with their corresponding AUC and 95% CI for mild-to-severe delay.

At the individual level, children with positive TOY8 results were 2.32 times more likely to experience delay. The Speech-Language domain showed the strongest discriminative power, with LR+ values of 5.34 and 7.19 for mild-to-moderate and severe delays, respectively, indicating a moderately high probability of true delay. Predictive and negative predictive values were not reported as they depend on prevalence. ROC analysis (Figure 1) showed excellent discrimination for the Cognitive, Speech-Language, and Fine Motor domains (AUC 0.82–0.84). Personal-Adaptive Behavior and Gross Motor were acceptable (AUC 0.74 and 0.71). For severe delays, AUCs were similar or slightly higher. Notably, TOY8 showed the strongest correspondence with the Griffiths III in the Cognitive, Speech-Language, and Fine Motor domains, which align closely with the Griffiths III Foundations of Learning, Language and Communication, and Eye-Hand Coordination subscales.

While an initial threshold of 40 was used, ROC analysis indicated that optimal cut-offs varied by domain (44–50). These refinements improved accuracy by reducing false negatives (missed cases) and false positives (unnecessary referrals). Optimal domain-specific cut-offs and corresponding sensitivity/specificity are presented in Table 4.

**Table 4:** Optimal cut-off for TOY8 screening tool subscales and the corresponding sensitivity, specificity and accuracy.

Developmental subscales	Optimal threshold	Sensitivity	Accuracy^a^	Specificity
Cognitive	45	0.82	0.69	0.62
Speech-language	49	0.81	0.76	0.64
Fine motor	44	0.83	0.69	0.64
Personal-adaptive behavior	50	0.73	0.66	0.62
Gross motor	50	0.78	0.67	0.62

a Fraction of correctly classified cases.

Parental perceptions of TOY8 were highly positive. Most rated it easy to use [34.1% (*n* = 44) very easy; 64.3% (*n* = 83) easy] and useful for detecting developmental issues [50.4% (*n* = 65) very useful; 48.8% (*n* = 63) useful]. Acceptability was high, with 96.9% agreeing that TOY8 was suitable for screening. Only two parents (1.6%) found it difficult, one (0.8%) not useful, and four (3.1%) did not find it acceptable. Perceptions did not differ significantly by parent education, gender, or ethnicity (*p* > 0.05).

## Discussion

This study presents the first validation of a digital developmental screening tool in Malaysia, demonstrating that TOY8 can accurately identify delays among children aged 3–5 years. TOY8 performed exceptionally well in detecting cognitive, speech-language, and fine motor delays, with AUCs ranging from 0.82 to 0.84. These domains are crucial because deficits in cognitive and speech-language development strongly predict later academic achievement and social adjustment. At the same time, fine motor skills underpin school readiness tasks such as writing, drawing, and self-care. Not an unexpected yet important finding was that ten children without a history of delay were later identified with developmental disorders on Griffiths III. Although the study was not designed to estimate the prevalence of undetected difficulties, this observation underscores the shortcomings of current Malaysian surveillance practices, which often end after 18 months. TOY8 may therefore be useful in identifying subtle or previously unrecognized concerns in preschoolers, enabling earlier referral and intervention.

Language delay is one of the most common developmental concerns, affecting up to 20% of preschoolers, and even higher proportions in disadvantaged groups. (26–28) Persistent language impairment is associated with long-term academic difficulties, including reduced literacy, comprehension, and increased grade repetition (29–31). Similarly, fine motor delays contribute to challenges in handwriting, mathematics, and self-care, which impair school readiness (32, 33). Conversely, higher levels of executive function and fine motor ability predict stronger kindergarten performance (34). Thus, a tool like TOY8, sensitive to these critical domains, addresses an important gap in early identification.

TOY8 appeared to be less sensitive in detecting gross motor and personal-social delays, though specificity remained high (0.82–0.84). Several factors explain this. First, the study excluded children with severe developmental disabilities, including those with significant physical limitations or hyperactivity, leading to more conservative estimates of accuracy in these domains. Second, gross motor delays in preschoolers are often subtle, involving coordination or motor planning rather than fundamental motor skills, and are less detectable through parent report than through professional observation (35). To enhance detection, future versions of TOY8 could integrate brief video-based motion-capture modules or combine screening with direct observational assessments such as the Test of Gross Motor Development, or teacher- or clinician-administered tools like the Movement Assessment Battery for Children—Second Edition (Movement ABC-2) Checklist and the Developmental Coordination Disorder Questionnaire (DCDQ). These complementary approaches emphasize the importance of multimethod assessment to identify and describe motor difficulties in young children, although each tool has its own practical limitations (35, 36). Likewise, personal-social skills are complex, context-dependent, and difficult to capture solely via parental questionnaires. The limitations of parent-reported tools are well-documented: they exhibit variable sensitivity, can diverge from direct assessments, and are influenced by caregiver perceptions (20, 37, 38). Incorporating refined cut-offs for both domains, or supplementing TOY8 with additional validated social-behavioral questionnaires, may strengthen detection in this area while maintaining feasibility for community-based use. This reflects broader evidence that the real-world accuracy of screening tools remains variable across settings and requires ongoing adaptation (39). Despite these limitations, TOY8 and Griffiths III assess overlapping constructs, with TOY8's digital, play-based design providing a scalable alternative for community-based screening.

Malaysia's multilingual context adds complexity. Most children are exposed to more than one language at home and preschool, making monolingual development uncommon. For example, it is not unusual for a Malay family that uses Malay dialects at home, and children are exposed to English and formal Malay language when they attend preschool. Similarly, for Chinese or Iban children, they might use the Chinese language (or one of the Chinese dialects) or Iban (or another Sarawak native language), respectively, at home. When the children attend preschool, they would all be exposed to English and the national language (i.e., Malay). Such diversity can influence both performance and parental reporting. This study did not systematically record children's primary and secondary languages, which is a limitation. Although some inferences can be drawn from speech-language domain performance, they do not fully capture the complexities of the multilingual environment. Future studies should examine the language environment in detail, though TOY8's bilingual design (Malay and English) already reflects local realities.

ROC analysis underscored the importance of domain-specific cut-offs rather than a single threshold. While an initial cut-off score of 40 was proposed, optimal thresholds varied by domain (44–50), improving sensitivity without major loss of specificity. Since the costs of missed cases generally outweigh those of false positives, higher sensitivity is desirable in screening contexts. These refined cut-offs provide a practical, evidence-based guide for referrals.

Parents rated TOY8 very positively, reporting high ease of use, usefulness, and acceptability. These findings are important, as parental acceptance influences both uptake and sustainability of screening programs, increasing the chances of timely detection and intervention. Positive parental engagement also supports collaboration with teachers and healthcare providers, enhancing developmental support networks for children.

Beyond its clinical implications, TOY8 has the potential to contribute to global child health and equity agendas (10). It supports SDG 3 by ensuring early detection of developmental delays and promoting well-being from the preschool years (11). Its scalability and bilingual design also address SDG 10 by reducing inequalities in access to developmental screening across diverse communities (11). Given the close links between child development, nutrition, and educational attainment, TOY8 indirectly supports SDG 2, which aims to end hunger and promote the growth and development of children (11).

The study has several strengths. Its case-control design with age- and sex-matching, use of Griffiths III as a gold standard, and bilingual format ensured methodological rigor and cultural relevance. Together, these features enhance the robustness of the findings and support the tool's cultural adaptability within Malaysia. TOY8's strong performance in speech-language, cognition, and fine motor detection addresses the critical preschool window highlighted by international guidelines. The American Academy of Pediatrics emphasizes continued surveillance between ages 3 and 5, when conditions like language disorder, autism, ADHD, and learning disabilities often emerge (3). TOY8's feasibility in community and preschool settings offers a solution to this widely recognized gap.

However, the study sample was confined to Sarawak and overrepresented Chinese participants in the control group, which may affect generalizability. Children with severe motor or sensory impairments were excluded, which restricted the applicability to this subgroup, although such children are typically identified earlier during routine MCH visits. The lack of systematic language environment data also limited the interpretation of language outcomes. Future work should validate TOY8 across different Malaysian regions and ethnic groups, in longitudinal cohorts, and in real-world preschool settings.

Despite these limitations, TOY8 shows important advantages. It demonstrated accuracy in core developmental domains, required minimal training, and was time-efficient. These features make it scalable in community and preschool settings where specialists are scarce. An additional strength is TOY8's integration with a digital intervention program, offering immediate, customized support for parents and teachers. This positions TOY8 not only as a screening tool but also as part of a broader digital ecosystem for early identification and intervention (8)..

### Implications for policy and practice

TOY8 has significant potential for integration into early childhood and public health systems. Its short administration time and ease of use by trained non-specialists enable screening to take place in preschools and community health services, where children naturally spend time, extending reach beyond hospital-based care. Embedding TOY8 within national maternal and child health programs and preschool policies could strengthen developmental surveillance during the critical 3- to 5-year gap before school entry, with bilingual availability enhancing equity across Malaysia's diverse communities (3). Earlier and more efficient identification of at-risk children can reduce waiting times and optimize the use of scarce specialist resources. Importantly, TOY8 is designed to complement, not replace, comprehensive assessment tools such as the Griffiths III, serving as a scalable first-line screener to prioritize children for detailed evaluation. As a smartphone-based tool, it can integrate into digital health ecosystems, facilitating longitudinal monitoring, data aggregation, and evidence-informed planning. Finally, the domain-specific cut-offs identified in this study provide refined referral thresholds that balance sensitivity with health system capacity.

### Future research directions

This study provides strong initial validation of TOY8, but further work is needed. Longitudinal studies should assess whether children identified through TOY8 benefit from earlier referral and intervention, and whether these translate into improved school readiness and long-term outcomes. The tool's digital design also allows integration with personalized learning and parent-guided activities. Ultimately, research on international adaptation, particularly in LMIC, could expand its role as a culturally accessible and adaptable tool for developmental screening.

## Conclusion

TOY8 is the first digital developmental screening tool formally validated in Malaysia, demonstrating good performance in detecting cognitive, speech-language, and fine motor delays—domains most predictive of later outcomes. Its short administration time, ease of use, and high parental acceptability support feasibility for community deployment. Although refinement is needed for the gross motor and personal-adaptive domains, TOY8 represents a culturally adapted and scalable solution.

## Data Availability

The original contributions presented in the study are included in the article/Supplementary Material, further inquiries can be directed to the corresponding author.
