# Supplementary material for: Case-control accuracy study for TOY8 digital developmental screening tool for detecting delays among children aged 3–5 years
Source: Front Pediatr. 2025 Dec 2;13:1706162. doi: 10.3389/fped.2025.1706162 (PMC12706852; doi:10.3389/fped.2025.1706162)
Supplement: Supplementary file 1 [file Supplementaryfile1.docx]

**Supplementary File**

**Sample size calculation**

In this study, the sample size (N) was estimated to achieve the expected precision in the estimates. High sensitivity was crucial for a developmental screening tool. It allowed parents, educators, and healthcare providers to identify young children with mild developmental delays for early intervention.

Sensitivity was defined as the probability of detecting true positives. Specificity was defined as the probability of detecting true negatives. We expected the index screening tool to achieve 80% sensitivity and specificity, equivalent to 80% accuracy. We also expected a +/-10% width (w) of 95% confidence interval (CI) of both sensitivity and specificity. In a case-control design, we did not consider the proportion of case (*p*) in the sample size calculation. In this study, we applied the sample size calculation method from Burderer et. al. 1996 [14].

First, we calculated the number of true positives + false negatives (TP+FN) for sensitivity and the number of true negatives + false positives (TN+FP) for specificity using the following equations, where the normal distribution value, *Z* was set to 1.96, corresponding with the 95% CI, the acceptable w of the 95% CI was set to 0.1 (10%), and the expected sensitivity and specificity were set to be 0.8 (80%).


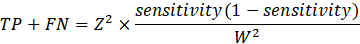
 (1)


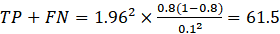
 (rounded to 62) (2)


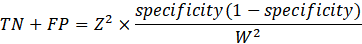
 (3)


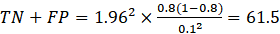
 (rounded to 62) (4)

The total sample size required = 62 cases + 62 controls = 124 subjects.

**Table A. Approximate distribution of cases and controls according to age and gender strata.**

| Overall | Study sample = 124 | | | |
| --- | --- | --- | --- | --- |
| Cohort | Cases = 62 | | Control = 62 | |
| Strata1: Gender | Male =31 | Female = 31 | Male = 31 | Female = 31 |
| Strata 2: Age | 3y = 10  4y = 11  5y = 10 | 3y = 10  4y = 11  5y = 10 | 3y = 10  4y = 11  5y = 10 | 3y = 10  4y = 11  5y = 10 |
